# Supplementary material for: Advancing the study of levels of best practice pre-service teacher education students from Spain: Associations with both positive and negative achievement-related experiences
Source: PLoS One. 2023 Jun 30;18(6):e0287916. doi: 10.1371/journal.pone.0287916 (PMC10313018; doi:10.1371/journal.pone.0287916)
Supplement: Attachment — Submitted filename: Response to Reviewers.docx [file pone.0287916.s001.docx]

**Manuscript ID**

**PONE-D-22-23792**

We thank the reviewers for his/her constructive comments and his/her thorough revision of the manuscript. Below we answer his/her questions and concerns, including explicitly the changes made in the manuscript as well.

**Musawenkosi Donia Saurombe, Ph.D.
Academic Editor
PLOS ONE**

**Journal Requirements:**

When submitting your revision, we need you to address these additional requirements.

1. Please ensure that your manuscript meets PLOS ONE's style requirements, including those for file naming. The PLOS ONE style templates can be found at

https://journals.plos.org/plosone/s/file?id=wjVg/PLOSOne_formatting_sample_main_body.pdf and

<https://journals.plos.org/plosone/s/file?id=ba62/PLOSOne_formatting_sample_title_authors_affiliations.pdf>.

- **Reponse:** Done

2. Peer review at PLOS ONE is not double-blinded (https://journals.plos.org/plosone/s/editorial-and-peer-review-process). For this reason, authors should include in the revised manuscript all the information removed for blind review.

- **Reponse**: Done

3. Please provide additional details regarding participant consent. In the ethics statement in the Methods and online submission information, please ensure that you have specified what type you obtained (for instance, written or verbal, and if verbal, how it was documented and witnessed). If your study included minors, state whether you obtained consent from parents or guardians. If the need for consent was waived by the ethics committee, please include this information.

- **Reponse**: The “written” consent has been specified in the procedure section.

4. Thank you for stating the following in the Acknowledgments Section of your manuscript:

“This research was funded by the XXXXXXXX PLAN FOR RESEARCH, DEVELOPMENT, AND INNOVATION (xxxxxxx; anonymized) OF THE xxxxxxxxxx (anonymized), grant number XXXXXXX (anonymized) (I+D+i research project)”.

We note that you have provided additional information within the Acknowledgements Section that is not currently declared in your Funding Statement. Please note that funding information should not appear in the Acknowledgments section or other areas of your manuscript. We will only publish funding information present in the Funding Statement section of the online submission form.

Please remove any funding-related text from the manuscript and let us know how you would like to update your Funding Statement. Currently, your Funding Statement reads as follows:

““This research was funded by the ANDALUSIAN PLAN FOR RESEARCH, DEVELOPMENT, AND INNOVATION (PAIDI 2020) OF THE JUNTA DE ANDALUCÍA, grant number P20_00148 (I+D+i research project)”.

Please include your amended statements within your cover letter; we will change the online submission form on your behalf.

- **Reponse:** Information pertaining to our source of funding has been removed from the manuscript, and instead this information has been added in the cover letter (re-submission).

5. Please include your full ethics statement in the ‘Methods’ section of your manuscript file. In your statement, please include the full name of the IRB or ethics committee who approved or waived your study, as well as whether or not you obtained informed written or verbal consent. If consent was waived for your study, please include this information in your statement as well.

- **Reponse**: This information has been included.

6. Please review your reference list to ensure that it is complete and correct. If you have cited papers that have been retracted, please include the rationale for doing so in the manuscript text, or remove these references and replace them with relevant current references. Any changes to the reference list should be mentioned in the rebuttal letter that accompanies your revised manuscript. If you need to cite a retracted article, indicate the article’s retracted status in the References list and also include a citation and full reference for the retraction notice.

- **Reponse**: No correction is needed from us.

**Reviewers' comments:

Reviewer's Responses to Questions**

1. Is the manuscript technically sound, and do the data support the conclusions?

The manuscript must describe a technically sound piece of scientific research with data that supports the conclusions. Experiments must have been conducted rigorously, with appropriate controls, replication, and sample sizes. The conclusions must be drawn appropriately based on the data presented.

Reviewer #1: Yes

Reviewer #2: Yes

2. Has the statistical analysis been performed appropriately and rigorously?

Reviewer #1: Yes

Reviewer #2: Yes

3. Have the authors made all data underlying the findings in their manuscript fully available?

The [PLOS Data policy](http://www.plosone.org/static/policies.action#sharing) requires authors to make all data underlying the findings described in their manuscript fully available without restriction, with rare exception (please refer to the Data Availability Statement in the manuscript PDF file). The data should be provided as part of the manuscript or its supporting information, or deposited to a public repository. For example, in addition to summary statistics, the data points behind means, medians and variance measures should be available. If there are restrictions on publicly sharing data—e.g. participant privacy or use of data from a third party—those must be specified.

Reviewer #1: Yes

Reviewer #2: Yes

4. Is the manuscript presented in an intelligible fashion and written in standard English?

PLOS ONE does not copyedit accepted manuscripts, so the language in submitted articles must be clear, correct, and unambiguous. Any typographical or grammatical errors should be corrected at revision, so please note any specific errors here.

Reviewer #1: Yes

Reviewer #2: Yes

5. Review Comments to the Author

Please use the space provided to explain your answers to the questions above. You may also include additional comments for the author, including concerns about dual publication, research ethics, or publication ethics. (Please upload your review as an attachment if it exceeds 20,000 characters)

**Additional Comments to the Author**

**Reviewer – 1**

**Comment:** The title seems not to fully reflect the contents of the manuscript (Antecedents → +Δ(L1 – L2) → Adaptive Outcome) (antecedents as identified in the contents are not reflected in the title). If possible consider adding the context of the study to the title as well.

- **Response:** We have revised the title to reflect the context of our study, likewise sample characteristics. The new title is: Advancing the study of levels of best practice of pre-service teacher education students from Spain: Associations with both positive and negative achievement-related experiences

**Comment:** Clarifying and positioning the levels of best practice at the beginning of the study will help readers to follow through the study (Actual best practice L1, Optimal best practice L2)

- **Response:** To ensure that we do not repeat ourselves, we have added in one sentence in the introduction to allude readers to the concepts of ‘actual best’ and ‘optimal best’. The sentence is as follows:

“This reflective question, importantly, alludes readers to an important theoretical premise, namely, the notation of ‘Δ_(L1 – L2)_’, which depicts the relationship between ‘actual best’, denoted as L_1_, and ‘optimal best’, denoted as L_2_.”

**Comment:** Figure 2 shows a clear conceptual model of the study, the antecedents are identified, this clarifies the study better however the introduction section should clearly align with the title as well as the conceptual model.

- **Response:** To ensure that we do not repeat ourselves, we have added in two sentences to recap the focus of inquiry, which closely aligns to our conceptualization (i.e., Fig 2) in the Introduction Section. The sentences are:

“Overall, then, we conceptualize a theoretical model for examination, which seeks to explore and identify different types of antecedents that could impart contrasting effects on the two levels of best practice. Furthermore, as described earlier, a related issue for examination is the explanatory and predictive role of the two levels of best practice.”

**Comment:** Indicate how the three comparable and comparative antecedents were identified and why they were selected for this study.

- **Response:** We have now added in a paragraph to indicate why we have chosen self-concept, optimism, and pessimism as antecedents for examination. The paragraph is as follows:

“An examination of the educational psychology literature indicates that there are numerous psychological variables (e.g., self-efficacy for learning)[1-3] and psychosocial factors (e.g., the impact of the home environment)[4, 5], which could function as antecedents of future adaptive outcomes [6-9]. By all account, choosing which psychological variable and/or psychosocial factor to examine is a personal feat, coinciding with existing research development, theoretical premise(s), a researcher’s interest, etc. Our choosing of three psychological variables (i.e., self-concept, optimism, and pessimism) for undertaking, in this case, is based on three important factors: (i) clear and consistent evidence from existing research development, which indicates the predictive role of the psychological variable (e.g., the predictive effect of optimism on academic performance)[10], (ii) advancing knowledge and theoretical understanding of the theoretical premise of the process of human optimization [11-13], which contends that different types of psychological variables may help to optimize a person’s state of functioning, and (iii) intellectual curiosity to seek theoretical understanding into the comparative antecedent roles of the chosen psychological variables (e.g., optimism *versus* pessimism)”.

**Comment:** The data analysis and interpretation were adequately done and present interesting findings.

- **Response:** Thank you very much.

**Comment:** Theoretical contributions are clearly outlined; however, the practical contributions are not fully presented.

- **Response:** We have revised the manuscript to ensure that the practical contributions section is more explanatory. This refinement included two sub-headings, titled:

i. Educational Implications for Consideration
ii. Methodological Insights for Development

Furthermore, we have expanded the Educational Implications for Consideration Section to included the following:

“As conceptualized in Fig 2, optimal best practice may operate as a central variable, which in turn would mobilize, govern, and/or direct a person’s thought and behavioural patterns for effective and/or non-learning experiences. With this in mind, it is plausible for us to consider the following for educational (or non-educational) purposes:

i. Optimal best practice as an important source of information, which could operate to optimize and motivate a person’s thought and behavioural patterns [1-3]. This considered viewpoint contends that perhaps, one (e.g., a teacher) could encourage individuals to focus on striving to achieve optimal best practice. Optimal best practice is personal and subject to individual variations between individuals, reflecting their differing levels of optimism, motivation, aspiration, positive outlook, etc. Striving to achieve optimal best practice, which is a personal best goal for accomplishment [8], may help motivate individuals to engage in the learning and/or non-learning processes. Within the context of academic learning in a specific subject or a variety of subjects, it is plausible for educators to use a psychological concept known as personal resolve [5, 6, 21], which we cited earlier, to encourage, promote, and/or facilitate optimal best practice. Personal resolve, in brief, is defined as a person’s “internal state of decisiveness and resolute to strive for optimal achievement best in an optimistic manner” [84]. As such, from this definition, personal resolve emphasizes the importance of a student’s self-determination to overcome any obstacles that may arise, guiding and directing her to engage in a purposive act to achieve optimal best (i.e., personal resolve → L_2_)[21, 84]. In this sense, personal resolve is analogous to an internal state of mental fortitude, serving as a potent source of motivation for learning and non-learning purposes. Timely use of positive feedback in class, for example, may instil and/or encourage a heightened state of personal resolve, which in turn could motivate a student to strive for optimal best practice.
ii. Design pathways, means, and/or opportunities, which could stimulate motivation for achievement of optimal best practice. Our present findings, for example, indicate support for the potential use of academic self-concept [e.g., 35, 36, 37], optimism [e.g., 33, 42, 43, 44], and other positive constructs (e.g., hope) [85-87] that could, indeed, serve to encourage and/or to stimulate the striving of optimal best. For example, positive verbal discourse (e.g., encouraging feedback, which may reflect a state of hope and inspiration) may instil a level of academic self-concept for learning, in turn helping to motivate students to strive for optimal best experiences. In a similar vein, encouraging a student to show optimism regardless of perceived difficulties, hardships, etc. could also help.

Interesting subject contents and/or contextual matters that have daily relevance, likewise, may assist to encourage individuals to strive to achieve optimal best practice. In terms of academic learning, for example, interesting subject contents (e.g., the study of paranormal psychology)[88] may encourage situational interest, intellectual curiosity, and aspiration for mastery, all of which could motivate a student to strive to achieve optimal best experiences. Difficult and/or uninterest subject contents (e.g., the learning of algebra)[20] that are perceived as being ‘boring’ [89], in contrast, are more likely to demotivate, weakening one’s interest, intellectual curiosity, aspiration to strive to achieve mastery. In a similar vein, a contextual matter or situation that has life relevance and/or daily applicability (e.g., the practice of meditation)[90-92] is more likely to enhance, stimulate, and/or facilitate, motivating and guiding a person to strive for optimal life functioning”.

**Comment:** Align the conclusions to the three major tenets identified under the section conceptualization of a Theoretical Model for Examination (line 144 to 148). Providing a conclusion for each examined question would shade more light in the summary/conclusión.

- **Response:** We have now added a final paragraph to conclude our article. The paragraph, in particular, summarizes our study and its results in light of the three tenets raised in the conceptualization. The concluding paragraph is as follows:

“Overall, then, as a recap, our path analysis of university students’ responses to a suite of Likert-scale questionnaires yielded a number of interesting findings, which supported our original propositions – that: (i) academic self-concept, optimism, and pessimism predicted a state of L_2_, and that only academic self-concept and optimism predicted a state of L_1_, (ii) both L_1_ and L_2_ positively influenced proactive academic engagement, and (iii) in line with existing research development is the positive association between L_1_ and L_2_”.

**Reviewer - 2**

**Comment:** The manuscript is accepted but requires minor changes. The authors should consider avoiding pronouns and write in the third-party writing style. Manuscript needs editing in relation with references, especially intext citation. There should be consistent use of referencing style. The statistical analysis (i.e., descriptive statistics and correlations) is clear.

- **Response:** We have gone back and ensured that our in-text referencing, using PLoS One’s Referencing style, is correct. We also thank Reviewer 2 for his/her suggestion regarding third-person. However, in recent years, the APA 7^th^ Edition Manual has recommended the use of first-person; first-person, according to the APA Manual, is more engaging.

**References**

1.	Bandura A. Self-efficacy: Toward a unifying theory of behavioral change. Psychological Review. 1977;84(2):191-215. PubMed PMID: 847061.

2.	Bandura A. Self-efficacy: The exercise of control. New York: W. H. Freeman & Co; 1997.

3.	Bandura A. Self-efficacy in changing societies: Cambridge university press; 1995.

4.	McCartney K, Dearing E, Taylor BA, Bub KL. Quality child care supports the achievement of low-income children: Direct and indirect pathways through caregiving and the home environment. Journal of Applied Developmental Psychology. 2007;28(5-6):411-26. doi: 10.1016/j.appdev.2007.06.010. PubMed PMID: WOS:000250159800005.

5.	Muola JM. A study of the relationship between academic achievement motivation and home environment among standard eight pupils. Educational Research and Reviews. 2010;5(5):213-7.

6.	Phan HP, Ngu BH. Teaching, Learning and Psychology. Docklands, Melbourne: Oxford University Press; 2019 10^th^ November, 2015.

7.	Eggen P, Kauchak D. Educational psychology: Windows on classrooms. 9^th^ ed. Harlow, Essex: Pearson Education Limited; 2014.

8.	Moreno R. Educational psychology. Hoboken, NJ: John Wiley & Sons, Inc.; 2010.

9.	Ormrod JE. Educational psychology: Developing learners. 5^th^ ed. Upper Saddle River, NJ: Pearson Merrill Prentice Hall; 2006.

10.	Wu JH. Academic optimism and collective responsibility: An organizational model of the dynamics of student achievement. Asia Pacific Education Review. 2013;14(3):419-33. doi: 10.1007/s12564-013-9269-6. PubMed PMID: WOS:000323652700014.

11.	Fraillon J. Measuring student well-being in the context of Australian schooling: Discussion Paper. Carlton South, Victoria: The Australian Council for Research; 2004.

12.	Phan HP, Ngu BH, Yeung AS. Achieving optimal best: Instructional efficiency and the use of cognitive load theory in mathematical problem solving. Educational Psychology Review. 2017;29(4):667-92. doi: <http://10.1007/s10648-016-9373-3>. PubMed PMID: WOS:000414547000001.

13.	Phan HP, Ngu BH, Yeung AS. Optimization: In-depth examination and proposition. Frontiers in Psychology. 2019;10(Article 1398):1-16. doi: 10.3389/fpsyg.2019.01398.
